# Capsular Polysaccharide of* Mycoplasma ovipneumoniae* Induces Sheep Airway Epithelial Cell Apoptosis* via* ROS-Dependent JNK/P38 MAPK Pathways

**DOI:** 10.1155/2017/6175841

**Published:** 2017-03-07

**Authors:** Zhongjia Jiang, Fuyang Song, Yanan Li, Di Xue, Ning Zhao, Jiamei Zhang, Guangcun Deng, Min Li, Xiaoming Liu, Yujiong Wang

**Affiliations:** ^1^Key Laboratory of Ministry of Education for Conservation and Utilization of Special Biological Resources in the Western, Ningxia University, Yinchuan, Ningxia 750021, China; ^2^College of Life Science, Ningxia University, Yinchuan, Ningxia 750021, China; ^3^Ningxia Key Laboratory of Clinical and Pathogenic Microbiology, the General Hospital of Ningxia Medical University, Yinchuan, Ningxia 750004, China

## Abstract

In an attempt to better understand the pathogen-host interaction between invading* Mycoplasma ovipneumoniae* (*M. ovipneumoniae*) and sheep airway epithelial cells, biological effects and possible molecular mechanism of capsular polysaccharide of* M. ovipneumoniae* (CPS) in the induction of cell apoptosis were explored using sheep bronchial epithelial cells cultured in air-liquid interface (ALI). The CPS of* M. ovipneumoniae* was first isolated and purified. Results showed that CPS had a cytotoxic effect by disrupting the integrity of mitochondrial membrane, accompanied with an increase of reactive oxygen species and decrease of mitochondrial membrane potential (ΔΨ*m*). Of importance, the CPS exhibited an ability to induce caspase-dependent cell apoptosis via both intrinsic and extrinsic apoptotic pathways. Mechanistically, the CPS induced extrinsic cell apoptosis by upregulating FAS/FASL signaling proteins and cleaved-caspase-8 and promoted a ROS-dependent intrinsic cell apoptosis by activating a JNK and p38 signaling but not ERK1/2 signaling of mitogen-activated protein kinases (MAPK) pathways. These findings provide the first evidence that CPS of* M. ovipneumoniae* induces a caspase-dependent apoptosis via both intrinsic and extrinsic apoptotic pathways in sheep bronchial epithelial cells, which may be mainly attributed by a ROS-dependent JNK and p38 MAPK signaling pathways.

## 1. Introduction

*Mycoplasma ovipneumoniae (M. ovipneumoniae, MO)* has been identified as a pathogenic agent of chronic nonprogressive infection of pneumonia in sheep and goats [1–4]; the nature between a protective and a pathological host response of* MO* infection currently remains largely unknown. In this regard, the role of capsular polysaccharide (CPS), a major active component for cellular adherence, invasion, immune modulation, and virulence of* M. ovipneumoniae* remains undefined [5], although several lines of evidences have shown that* M. ovipneumoniae* is able to produce polysaccharide capsules for facilitating its adherence to ciliated epithelium [3, 6]. In this context, the respiratory epithelium is responsible for facilitating key defense mechanism and acting as first line of the immune system in response to a pathogen infection, including* M. ovipneumoniae,* for which tract epithelial cells serve as portals for bacteria entering hosts. Together with the pivotal role of CPS in the adherence of* M. ovipneumoniae* to host cells, it is therefore of importance to characterize biological functions and underlying mechanisms of immune responses of the CPS in respiratory epithelial cells.

Apoptosis is an active form of programmed cell death that plays a crucial role in the development and maintenance of organism homeostasis by eliminating of damaged or redundant cells [7–9]. In this context, organisms can employ antioxidant defense system to counteract oxidative stress and further prevent oxidative damage [10]. A compelling body of studies has indicated that reactive oxygen species (ROS), including H_2_O_2_, superoxide anion radical, and hydroxyl radical, contribute to the modulation of apoptosis signaling pathways [7]. Among them, an excessive ROS level is highly reactive and toxic and is likely to damage the biomacromolecules such as proteins, lipids, carbohydrates, and DNA [11, 12], which therefore leads to oxidative stress, which in turn triggers the activation of caspase cascades, subsequently inducing a cell apoptosis [13].

In the present study, we interrogated the biological activity and mechanism of capsular polysaccharides (CPS) of* M. ovipneumoniae-*induced respiratory epithelial cell apoptosis using air-liquid interface- (ALI-) cultured epithelial cells generated with primary bronchial epithelial cells of Tan sheep [14, 15]. The results showed that CPS of* M. ovipneumoniae* could induce sheep bronchial epithelial cell apoptosis through ROS-dependent JNK/P38 MAPK- but not ERK MAPK-mediated apoptotic pathways.

## 2. Materials and Methods

### 2.1. Reagents

The high glucose DMEM, trypsin, and penicillin-streptomycin solution were products of Hyclone Company (Logan, UT, USA). Bronchial epithelial cell Growth Medium (BEGM) was purchased from Lonza Group (Basel, Switzerland). Ultroser G (USG) medium was obtained from Pall Corporation (Washington, DC, USA). Fetal bovine serum (FBS) was purchased from Thermo Company (Rockford, MD, USA). Type I rat tail collagen and the Annexin V-FITC Apoptosis Detection Kit were purchased from BD Biosciences (San Jose, CA, USA). JC-1 (5,50,6,60-tetrachloro-1,10,3,30-tetraethylben zimidazolcarbocyanine iodide) and BCA protein assay kit were products of Keygen Biological Inc. (Nanjing, China). DCFH-DA (2′,7′-dichlorofluorescein diacetate) and DEAE-cellulose anion-exchange chromatography column were obtained from Sigma (St. Louis, MO, USA). Caspase-3, -8, and -9 Activity Assay Kit and LDH Cytotoxicity Assay Kit were purchased from Beyotime Company of Biotechnology (Shanghai, China). Chemicals SP600125, U0126, and SB203580 were purchased from MedChem Express (Hangzhou, China). Enhanced Western Bright ECL reagent was purchased from Advansta (Menlo Park, CA, USA). Antibodies against Bcl-xl, Bax, Bcl-2, Cytochrome c, apoptosis inducing factor (AIF), cleaved-caspase-3, CAT, SOD_2_, FADD, FAS, FASL, cleaved-PARP1, cleaved-caspase-8, ERK, and *β*-actin were products of Proteintech (Chicago, IL, USA). Antibodies to p-ERK, JNK, and P38 were produced by Signalway Antibody (Rockford, MD, USA). Antibodies to p-JNK, c-Jun, p-c-Jun, and P38 were obtained from Cell Signaling Technology (Beverly, MA, USA). The antibody to GSS was purchased from Abgent (San Diego, CA, USA). PCF Millicell inserts were purchased from Millipore (Bedford, MA, USA).

### 2.2. Propagation of* M. ovipneumoniae* and Purification of CPS

The* M. ovipneumoniae* Queensland Strain Y98 was grown and propagated in a* Mycoplasma* broth containing* Mycoplasma* broth base CM403, supplement-G SR59 (OXOID, Hampshire, UK) as described previously [14]. In order to maximize yield of polysaccharide, glucose was added in the culture medium with a final concentration of 10%; the* Mycoplasma* cells were cultured at 37°C for two or three days after the medium color was changed from red to yellow; the cells were then collected by centrifugation at 12,000*g* for 30 min at 4°C. The preparation of CPS was performed as described previously with modifications [6]. Briefly, the cell pellet was washed three times with phosphate buffered saline (PBS, pH = 7.4) containing 10% glucose prior to the isolation/extraction procedure, in order to minimize potential contaminants. Mycoplasma polysaccharides were extracted using 60°C preheated phenol for 30 min with constantly stirring. The resulting extracts in the aqueous phase were dialyzed in cellulose membrane tubing (exclusion limit 3500 Da), prior to be further treated with DNase I, RNase, and pronase K for removal of nucleic acids and protein contaminations, which was confirmed by a spectrophotometric assay in terms of determining *A*_260/280_ ratio. The extracts were then subjected to phenol/chloroform extraction (1 : 1) using a Phase-lock gel (Tiangen, Beijing, China), followed by being extracted with an equal volume of chloroform in order to remove possible lipophilic components of cytoplasmic membranes. The upper aqueous phase was subsequently collected and dialyzed against distilled water and PBS, followed by being precipitated with 4 equivalent volume of cold ethanol containing 3 M sodium acetate at 4°C overnight. The result was crude polysaccharides, which was dissolved in distilled water and then lyophilized using a vacuum freeze dryer. For purification of CPS, above crude CPS was dissolved in H_2_O and applied to a 1.0 by 5 cm DEAE-cellulose anion-exchange chromatography column, and fractions were eluted with a linear gradient of 0–1.0 M NaCl at a flow rate of 0.5 mL/min. Thirty-milliliter fractions were equally collected into 100 consecutive tubes. The content of polysaccharide of effluents was then detected by an improved phenol-sulfuric acid colorimetric method at 490 nm [16]. As a result, purified CPS-containing factions were collected, lyophilized, and stored at −80°C until further use.

### 2.3. In Vitro ALI Culture of Sheep Bronchial Epithelium and Infection of* M. ovipneumoniae*

This study was approved by the ethics committee for the care and use of animals at Ningxia University. Primary culture of sheep bronchial epithelial cells was prepared as previously reported in our lab [14]. Briefly, bronchial epithelial cells were obtained from 1.5- to 2.0-year-old* Tan* sheep. Bronchus was washed and digested overnight at 4°C using DMEM/F12 medium (1 : 1) containing 5% FBS and 0.1% DNaseI. Cells were then collected by centrifugation at 800 ×g for 5 min at 4°C. Subsequently, fibroblast cells were removed* via* adherence in basic media, while nonadherent cells were collected and cultivated in collagen-coated Millicell insert membrane using 5% FBS/BEGM medium at 37°C for 24–48 h. The BEGM was then replaced by Ultroser G medium (USG) (F12/MEM 1 : 1, +Supplements Mix +2% USG) to establish an air-liquid interface (ALI) culture system. After 4–6 weeks of cultivation, epithelial cells were well-differentiated. For a 2.4 cm diameter Millicell insert membrane, normally 1 × 10^7^ well-differentiated epithelial cells were determined [17]. For infection or treatment,* M. ovipneumoniae* cells or CPS were suspended or diluted in 2% USG medium and applied on the apical surface of ALI epithelial cells for infection with indicated time periods; equal volume of 2% USG medium was used as untreated control. Volumes of 0.5 mL and 0.1 mL were employed for covering wells with diameters of 2.4 cm and 1.2 cm, respectively.

### 2.4. Cell Viability (CCK) Assays and Lactate Dehydrogenase (LDH) Assays

Cell viability was determined by Cell Counting Kit-8 (CCK-8) assay according to the manufacturer's instruction. In brief, cells (5 × 10^3^ cells/well) were seeded in 96-well microplates and cultured overnight at 37°C. The cells were then treated with* M. ovipneumoniae* or CPS for indicated time periods. Subsequently, the CCK-8 solution (10 *μ*L) was added to each well and incubated for additional 4 h. Absorbance at 450 nm was measured using an ELISA reader (Bio-Rad Laboratories, Richmond, CA, USA). The death of ALI epithelial cells was determined by a LDH assay. Briefly, cells cultured in 1.2 cm diameter inserts were pretreated with or without N-acetyl-cysteine (NAC, ROS scavenger) (10 mM) for 2 h, followed by exposure to CPS (100 ng/mL),* MO* (multiplicity of infection, MOI = 30), or H_2_O_2_ (1 mmol/L) for 48 h. Media were used for analysis using a LDH Cytotoxicity Assay Kit according to the manufacturer's instruction. Subsequently, the absorbance at 450 nm was measured using a plate reader. Untreated ALI cells were served as a blank control. The cytotoxicity was determined according to the following formula: cytotoxicity (%) = OD (tested sample − control)/OD (positive control − control) × 100%. The positive control was provided in the kit.

### 2.5. Flow Cytometric Analysis of Apoptosis

The 4-week ALI cultures were infected with* MO* or treated with CPS as described above. The cultures were washed three times in PBS at 37°C prior to be disassociated with trypsin, subsequently harvested by centrifugation, and finally resuspended in 1x binding buffer (10 mM HEPES/NaOH, 140 mM, NaCl, 2.5 mM CaCl_2_, pH 7.4) Annexin V-FITC/propidium iodide (PI) double-staining assay was detected by flow cytometry using Apoptosis Detection Kit according to the manufacturer's instruction. The data was then analyzed using a flow cytometry system (CyFlow® Cube 15, USA).

### 2.6. Mitochondrial Membrane Potential (MMP) Assay

JC-1 is a common fluorescent cationic probe to detect the disruption of mitochondrial membrane potential. Mechanistically, JC-1 forms red fluorescence at high mitochondrial membrane potential, while green fluorescence indicates a lower membrane potential. Cells (1 × 10^7^ cells/well) were incubated with 10 *μ*M JC-1 staining at 37°C for 30 min and then the mitochondrial potential was detected using a CyFlow Cube 15 and analyzed by FCS Express 5.0 software.

### 2.7. Intracellular ROS Level Determined by DCFH-DA Assay

Intracellular ROS levels can be measured using 2′,7′-di-chlorodihydrofluorescein diacetate (DCFH-DA) following manufacturer's instruction. Briefly, 6-well ALI inserts were treated with/without NAC (10 mM) for 2 h, followed by exposure to CPS (100 ng/mL) or* MO* (MOI = 30) for 48 h. After rinsing with PBS twice, cells (1 × 10^7^ cells/well) were subsequently collected and treated in PBS containing 10 *μ*M DCFH-DA at 37°C for 20 min. Fluorescence intensity was measured at 488 nm/525 nm (excitation/emission wavelengths) by a CyFlow Cube 15 system. Data from at least three independent experiments were analyzed with FCS Express 5.0 software.

### 2.8. Caspase-3,-8, and -9 Activity Assay

The enzymatic activity of caspase-3, -8, and -9 were determined using caspase activity assay kit following manufacturer's instruction. Briefly, 6-well ALI inserts were infected with indicated conditions for 48 h. Cells were subsequently washed with cold PBS twice and lysed using 50 *μ*L of lysate buffer at 4°C overnight. After being centrifuged at 12,000 ×g for 10–15 min at 4°C, the supernatant was obtained. Protein concentrations were determined by the Bradford assay (BCA). Meanwhile, assay was performed using 96-well microplates by mixing 40 *μ*L of the supernatant (cytosolic extracts, 100 *μ*g), 50 *μ*L of lysis buffer, and 10 *μ*L of Ac-DEVD-pNA (a caspase-3 colorimetric substrate, N-acetyl-Asp-Glu-Val-Asp-P-nitroanilide), Ac-IETD-pNA (a caspase-8 colorimetric substrate, N-acetyl-Ile-Glu-Thr-Asp-P-nitroanilide), or Ac-LEHD-*p*NA (a caspase-9 colorimetric substrate, N-acetyl-Leu-Glu-His-Asp-P-nitroanilide) at 37°C overnight. Absorbance was measured in a plate reader at a wavelength of 405 nm (Bio-Rad Laboratories, Richmond, CA, USA).

### 2.9. Western Blotting Analysis

6-well ALI inserts were infected with indicated conditions for 48 h. Cells were washed with cold PBS twice and lysed in RIPA buffer containing protease/phosphatase inhibitors (Beyotime, Beijing, China) overnight at 4°C. Protein concentrations were then determined by BCA protein assay. The protein samples were resolved by 6–15% SDS-PAGE and subsequently transferred to polyvinylidene fluoride (PVDF) membranes (Millipore, Bedford, MA, USA). Membranes were blocked with 5% nonfat milk in TBST at room temperature for 2–4 h, followed by incubation with appropriate specific primary antibodies to proteins of interest overnight at 4°C. After extensive washing, membranes were probed with appropriate HRP-labelled secondary antibodies for 1-2 h at room temperature. Blots were finally developed and visualized using an ECL Advanced Western Blot Detection Kit. The specific protein blots were semiquantified by densitometric analysis using Image J software.

### 2.10. Statistical Analysis

All data in this study were expressed as the mean ± SD from data of at least three independent experiments. SPSS statistics 17.0 (SPSS Inc., Chicago, IL, USA) was used for the statistical analysis. Statistical differences between groups were performed using one-way analysis of variance (ANOVA) followed by post hoc Tukey's test. A *p* value < 0.05 was considered statistically significant.

## 3. Results

### 3.1. Isolation and Purification of CPS of* M. ovipneumoniae*

**Figure 1:**
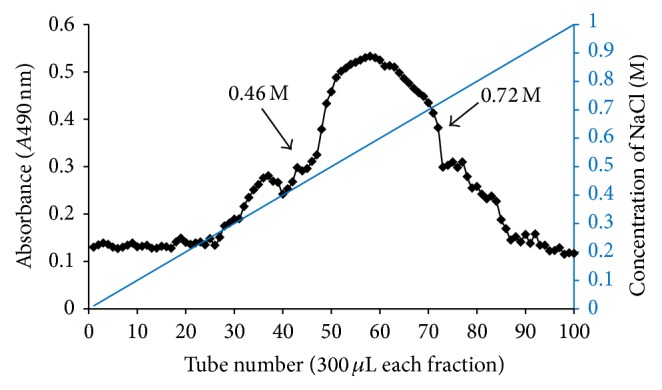
The purification of capsular polysaccharide from* M. ovipneumoniae* by HLPC. Elution curve of the capsular polysaccharide from* M. ovipneumoniae* by a DEAE-52 cellulose chromatography. The column was first eluted with distilled water, followed by eluting with a linear concentration gradient of NaCl from 0 to 1.0 mol/L.

Phenol and ethanol have been commonly used for the extraction of crude polysaccharides. In the present study, the crude capsular polysaccharide of* M. ovipneumoniae* has been successfully isolated by a phenol extraction and ethanol precipitation method. The average yield of crude capsular polysaccharides was 4.658 ± 0.662% of wet weight of bacteria. There were no detectable nucleic acid and protein contamination as determined by a ratio of absorption at 260 and 280 nm. To obtain high-purity of polysaccharide products for biofunctional studies, a subsequent purification was performed by a high performance liquid chromatography (HPLC) using a cellulose DEAE-52 column.  Figure 1 showed the curve of capsular polysaccharide detected by an improved phenol-sulfuric acid colorimetric method [16]. The curve displayed a main peak of CPS between fractions 46 to 72 following a linear elution. The eluents of this peak were collected and lyophilized. The average recovery rate of the purified CPS was 62.26 ± 5.002% of the crude CPS in this study. This purified CPS was used for subsequent biofunctional analysis.

### 3.2. Effects of CPS on Cell Viability in ALI Cultures of Sheep Bronchial Epithelial Cells

**Figure 2:**
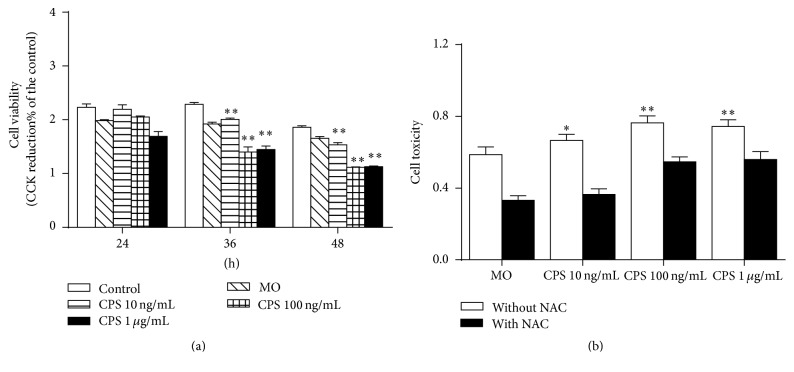
Impacts of CPS on cell viability of sheep bronchial epithelial cells. (a) Cell viability treated with various concentrations of CPS was measured by a CCK assay after 24, 36, and 48 h treatment. (b) 4-week-old ALI cultures of sheep bronchial epithelial cells were apically treated with capsular polysaccharide (CPS) at 100 ng/mL or infected with* M. ovipneumoniae* (MO) at MOI of 30 for 48 h before samples were harvested for analysis. Cells were pretreated with/without NAC (10 mM) for 2 h, followed by exposure to indicated conditions for 48 h. Results showed that LDH release was significantly increased versus the control group and that it can be reversed by NAC. Values are mean ± SD for at least three independent experiments performed in triplicate. ^*∗*^*p* < 0.05 and ^*∗∗*^*p* < 0.01 versus control.

In order to evaluate biological effects of CPS and* M. ovipneumoniae* in sheep bronchial epithelial cells, their effect on cell viability was first determined by a Cell Counting Kit-8 (CCK-8) assay. Results showed that both CPS and* M. ovipneumoniae* significantly inhibited the cell proliferation in a time- and dose-dependent manner, with an exception of no difference between cells exposed to 100 ng/ml and 1 *μ*g/ml of CPS for 36 h and 48 h, respectively (Figure 2(a)). In agreement with results of the CCK assay, the LDH assay showed a similar result, and there was no significant statistical difference between cells treated with 100 ng/ml and 1.0 *μ*g/ml of CPS for 48 h (Figure 2(b)). Moreover, to assess whether the CPS-induced cell death was dependent on ROS production, cells were pretreated with NAC, a ROS scavenger prior to CPS treatment. The result showed pretreatment with NAC significantly suppressed both CPS- and* MO-*mediated cytotoxicity, suggesting that ROS might play key roles involved in CPS- and* MO-*induced cell death (Figure 2(b)). As the preferable effect of CPS on cell viability was observed at 100 ng/mL, the concentration of 100 ng/mL CPS was chosen for the following experiments.

### 3.3. CPS-Induced Cell Apoptosis in ALI Cultures of Sheep Bronchial Epithelial Cells

**Figure 3:**
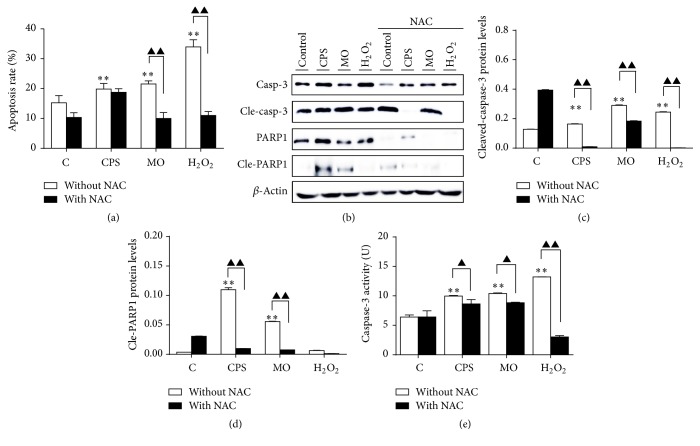
CPS-induced apoptosis in ALI cultures of sheep bronchial epithelial cells. Cells were pretreated with/without NAC (10 mM) for 2 h, followed by exposure to indicated conditions for 48 h. (a) 4-week-old ALI cultures of sheep bronchial epithelial cells were apically infected with CPS at 100 ng/ml and* MO* at MOI of 30 for 48 h before samples were harvested for analysis. Percentages of apoptotic cells were determined by flow cytometry using Annexin V/PI double-staining assay. (b) Immunoblots of apoptosis-associated proteins. The blots were probed for *β*-actin as a loading control. (c and d) Representative blots for cleaved-caspase-3 and cleaved-PARP1 were semiquantified by a densitometric analysis by calculating the fold of change of a protein of interest over *β*-actin. (e) Relative caspase-3 activity of ALI cells was detected after treating with indicated conditions. Values are mean ± SD for at least three independent experiments performed in triplicate. Compared to non-CPS or MO treatment, ^*∗*^*p* < 0.05 and ^*∗∗*^*p* < 01. Comparing between indicated groups, ^▲^*p* < 0.05 and ^▲▲^*p* < 0.01.

Apoptosis is a process of programmed cell death that occurs in multicellular organisms [7]. In present study, a flow cytometry assay following Annexin V/PI double-staining was used to evaluate whether CPS- and* MO-*induced cell deaths were relevant to apoptosis. As expected, a significantly increased apoptotic cell fraction was observed in cells treated with CPS or* M. ovipneumoniae* infection compared to untreated cells (Figure 3(a)). The percentage of CPS-induced apoptotic cells was increased from 15.26% to 19.86%, while the fraction of* M. ovipneumoniae*-treated apoptotic cells rose to 21.54%, suggesting that both CPS and* MO* remarkably induced a cell apoptosis progression. Unexpectedly, it is worth noting that pretreatment with NAC could not apparently inhibit the CPS-induced cell apoptosis, although NAC could dramatically alleviate cell apoptosis induced by a* M. ovipneumoniae* infection. These results indicated that the CPS-induced cell apoptosis might be not mainly attributed by ROS production. As caspase-3 and its downstream substrate, PARP, play an active role in the early apoptotic events, and a cleaved form of PARP has been considered as an indicative of functional caspase activation [18]. Western blot performed on the cell lysates obtained from CPS- and* M. ovipneumoniae-*treated cells showed an increased expression of cleaved-caspase-3 and the active form of PARP (Figures 3(b)–3(d)). As expected, exposure of ALI cells to CPS and* MO* for 48 h resulted in a 1.56-fold and 1.62-fold increase of caspase-3 activity when compared with untreated controls, respectively. Moreover, cells pretreated with NAC exhibited a decrease in the CPS- and* MO-*induced caspase-3 activation, therefore indicating the involvement of caspase-dependent apoptosis (Figure 3(e)).

### 3.4. ROS Generation and Oxidative Stress Induced by CPS in ALI Cultures of Sheep Bronchial Epithelial Cells

**Figure 4:**
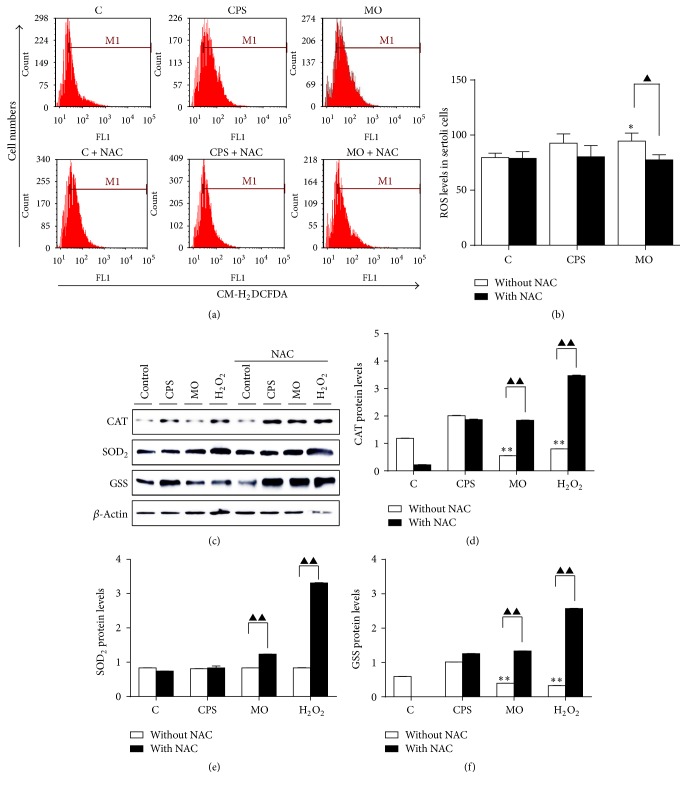
The CPS-induced oxidative stress in ALI cultures of sheep bronchial epithelial cells. (a and b) Scavenging of CPS- and* MO-*induced ROS by NAC. Cells were pretreated with/without NAC (10 mM) for 2 h, followed by exposure to indicated conditions for 48 h. Intracellular ROS were measured by DCF fluorescence after 10 *μ*M DCFH-DA staining using flow cytometry. Geometric mean of fluorescence intensity values was calculated and compared to that in control. (c) Expression levels of REDOX-associated proteins. *β*-actin was used as the loading control. Immunoblots showed here were a representative of three independent experiments with similar results. (d–f) Densitometric values were shown for CAT, SOD_2_, and GSS proteins over *β*-actin. Values were mean ± SD for at least three independent experiments performed in triplicate. ^*∗*^*p* < 0.05 and ^*∗∗*^*p* < 0.01 versus control. Compared between indicated groups, ^▲^*p* < 0.05 and ^▲▲^*p* < 0.01.

**Figure 5:**
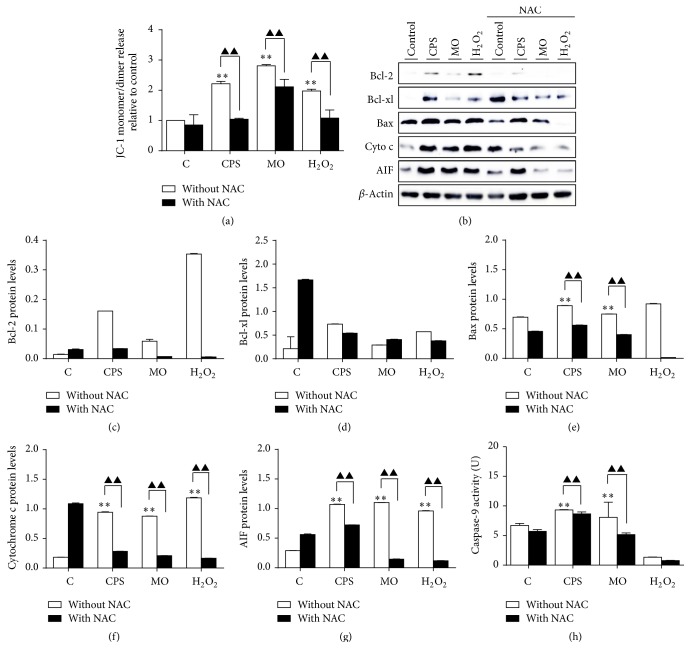
Impacts of CPS on the mitochondrial apoptosis pathway in ALI cultures of sheep bronchial epithelial cells. Cells were pretreated with/without NAC (10 mM) for 2 h, followed by exposure to indicated conditions for 48 h. (a) Changes of ΔΨ*m* on ALI cells were detected by JC-1 staining and analyzed by flow cytometry. Cells with high ΔΨ*m* exhibit red fluorescence, while cells with low ΔΨ*m* exhibited green fluorescence. Quantitative analysis of the shift of mitochondrial red fluorescence to green fluorescence among groups was detected. The ratios of green fluorescence intensity to red fluorescence intensity were calculated. Cells treated with USG medium alone served as control. (b) Western blotting of mitochondrial apoptosis-related proteins. The blots were probed for *β*-actin as a loading control. Immunoblots shown here were representatives of three independent experiments with comparative results. (c–g) Densitometric analysis of Western blots showed Bcl-2, Bcl-xl, Bax, cytochrome c, and AIF expression over *β*-actin in ALI cells. (h) Relative caspase-9 activity of ALI cells treated with indicated conditions was ascertained using a kit. Values are mean ± SD for at least three independent experiments performed in triplicate. Compared to non-CPS or MO treated control, ^*∗*^*p* < 0.05 and ^*∗∗*^*p* < 0.01; compared to non-NAC treated group, ^▲^*p* < 0.05 and ^▲▲^*p* < 0.01.

There is now widespread and strong evidence implicating that ROS and oxidative stress play key roles in mediating the mitochondrial dysfunction [19, 20]. Accumulation of excessive ROS can directly interact with cellular components, such as DNA and protein, thereby causing oxidative stress and subsequent cell death [13]. With this in mind, we further investigated whether the elevated ROS level of CPS- or* MO*-treated cells served as a cause of apoptosis induced by these insults. FACS analysis revealed that both CPS and* M. ovipneumoniae* could induce a notably increased ROS production compared to controls, as measured by an oxidant-sensitive probe, CM-H_2_DCFDA. These effects were almost completely abolished by scavenging ROS with NAC, indicative of a potential role of CPS and* M. ovipneumoniae* in ROS production in sheep bronchial epithelial cells (Figures 4(a) and 4(b)). Protective antioxidants form the first line of defense against ROS by reacting directly with free radicals, or by enhancing the bioactivity and expression of various antioxidant enzymes, such as superoxide dismutase (SOD), catalase (CAT), and glutathione synthetase (GSS) [21]. With this in mind, to further explore whether CPS could induce oxidative stress in ALI cells, we performed Western blots to examine the activities of SOD, CAT, and GSS. Results showed an unexpectedly upregulated expression of CAT and GSS in cells treated with CPS, except that the SOD_2_ level was slightly decreased in this group compared to the vehicle control (Figures 5(d)–5(f)). In addition, results also exhibited that an infection of* M. ovipneumoniae* could decrease the abundance of above three proteins. In contrast, a pretreatment of NAC could restore the expression of all the three redox-related proteins suppressed by* M. ovipneumoniae*. These results thus might indicate that an oxidative stress might contribute to the* M. ovipneumoniae*-induced cell apoptosis.

### 3.5. CPS Triggered an Intrinsic Apoptotic Pathway in Sheep Bronchial Epithelial Cells

Apoptosis is a highly regulated and controlled process, which can be initiated through two pathways: mitochondria-mediated (intrinsic) and cell-death-receptor-mediated (extrinsic) apoptotic pathways [7, 22]. To further interrogate the mechanism underpinning processes of CPS-induced apoptosis, several apoptosis-associated parameters widely implicated in these two pathways were examined. The cell membrane potential (ΔΨ*m*) is a key index to evaluate the integrity of mitochondrial membrane. Disruption of ΔΨ*m* by several factors, for instance, excessive ROS generation, can cause cell damage [23]. JC-1, a fluorescent probe, was thus applied to observe whether there was loss in ΔΨ*m* using a flow cytometry-based assay. Indeed, an exposure to CPS or* M. ovipneumoniae* alone for 48 h resulted in an evident elevation in percentages of cells with green fluorescence to more than 200% as compared with the control (Figure 5(a)), indicating a moderate decrease of ΔΨ*m* in treated cells. Notably, a pretreatment of NAC almost completely or partly reversed the loss of ΔΨ*m* induced by CPS exposure or* M. ovipneumoniae* infection. Molecular analysis by Western blotting assay further revealed an upregulated proapoptotic protein Bax, AIF, and cytochrome c (Figures 5(b)–5(g)). Consistently, the increased expression of proapoptotic proteins could be partially blocked by pretreatment of NAC (Figures 5(b)–5(g)). Meanwhile, an enhanced activity of caspase-9, an initiator caspase linking to the intrinsic apoptotic pathway, was also observed (Figure 5(h)). This finding suggested that pretreatment with NAC could alleviate CPS or bacteria-induced intrinsic apoptosis in part, indicating that a ROS-dependent mitochondrial-mediated (intrinsic) apoptosis might be involved in the CPS- and* M. ovipneumoniae-*induced apoptosis in sheep bronchial epithelial cells.

### 3.6. CPS Triggered an Extrinsic Apoptotic Pathway in Sheep Bronchial Epithelial Cells

**Figure 6:**
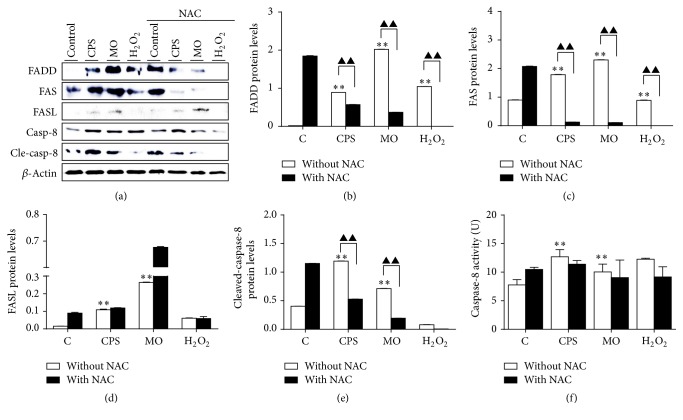
Effects of CPS on cell-death-receptor-mediated (extrinsic) apoptotic pathway in ALI cultures of sheep bronchial epithelial cells. Cells were pretreated with/without NAC (10 mM) for 2 h, followed by exposure to indicated conditions for 48 h. (a) Expression of FAS-associated apoptosis-related proteins. The protein levels of FADD, FAS, FASL, caspase-8, and cleaved-caspase-8 were detected by Western blotting assay. Immunoblots shown here were representative of three independent experiments with comparative results. (b–e) Semiquantitative analysis of immunoblots shown in (a). *β*-actin was used as an internal control and a semiquantification was performed by a densitometric analysis by calculating the fold of change of a protein of interest over *β*-actin. (f) Relative caspase-8 activity was detected after treating with indicated conditions. Values are mean ± SD for at least three independent experiments performed in triplicate. Compared to non-CPS or MO treated control, ^*∗*^*p* < 0.05 and ^*∗∗*^*p* < 0.01; compared to non-NAC treated group, ^▲^*p* < 0.05 and ^▲▲^*p* < 0.01.

We next sought to investigate whether an extrinsic apoptotic pathway was involved in the cell apoptosis induced by CPS or bacteria cells of* M. ovipneumoniae.* Apoptosis can be initiated by ligation of death receptors on cell surface to their cognate death receptor ligands. It is well known that FAS-associated death domain (FADD) protein is a key adaptor in the death-receptor-mediated apoptosis [24]. In this context, the death receptor FAS is activated upon the binding of FAS ligand (FASL) and cytoplasmic FADD* via* its death domain (DD), which in turn further recruits and activates procaspase-8 to initiate an induction of apoptosis [24, 25]. To assess an involvement of extrinsic signaling in CPS- or* M. ovipneumoniae-*induced cell apoptosis, the expression of FAS/FASL signaling-related proteins, including FADD, FAS, FASL, and cleaved-caspase-8 was examined. Of interest, a significant upregulation of above extrinsic apoptotic proteins was determined in both CPS- and* M. ovipneumoniae*-treated cells, and a pretreatment of NAC led to almost completely abolishing of the induced expression of these proteins, particularly the cleaved form of caspase-8, except the FASL, which was slightly increased in the CPS-treated group (Figures 6(a)–6(e)). In consistence, biological activity assays further confirmed an enhanced activation of caspase-8 activity in the CPS or* M. ovipneumoniae-*treated cells, which could be inhibited by NAC (Figure 6(f)). Taken together, these data clearly suggest that the extrinsic apoptotic signaling is also involved in the CPS/*M. ovipneumoniae*-induced apoptosis in sheep bronchial epithelial cells.

### 3.7. Activation of the MAPK Signaling Pathway in Sheep Bronchial Epithelial Cells in Response to CPS Stimulation

**Figure 7:**
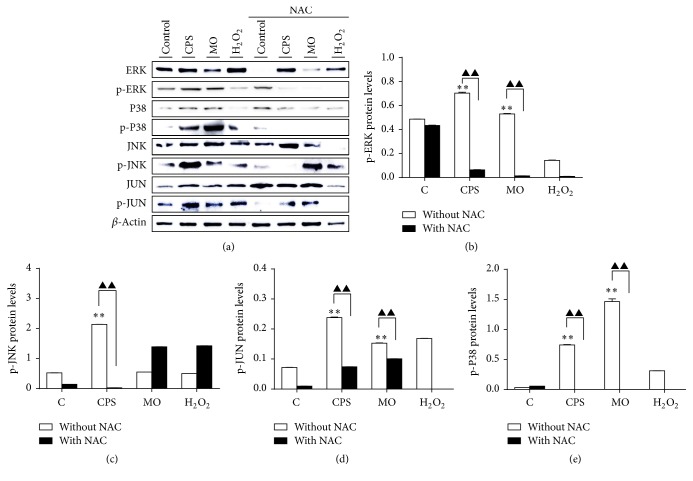
CPS-activated ERK1/2, JNK, and P38 signaling of MAPK pathways in ALI cultures of sheep bronchial epithelial cells. Cells were pretreated with/without NAC (10 mM) for 2 h, followed by exposure to indicated conditions for 48 h. (a) Total cell lysates were subjected to Western blotting analysis using indicated antibodies. The blots were probed for *β*-actin as a loading control. Results showed that NAC significantly suppressed CPS-induced phosphorylation of ERK1/2, JNK/c-Jun, and P38. (b–e) Protein ratio of interest. protein/*β*-actin was calculated following ImageJ densitometric analysis. These bar graphs shown here were representative of three independent experiments with similar results. Compared to non-CPS or MO treated control, ^*∗*^*p* < 0.05 and ^*∗∗*^*p* < 0.01; compared to non-NAC treated group, ^▲^*p* < 0.05 and ^▲▲^*p* < 0.01.

In order to explore a potential mechanism underlying CPS-induced cell apoptosis, we next examined the activation of mitogen-activated protein kinase (MAPK) signaling in sheep epithelial cells, since evidence has recently revealed that MAPK signaling acts as a key signal transduction pathway in regulating cell inflammation and apoptosis. To this end, ALI cultures were pretreated with NAC for 2 h, followed by exposing to CPS or* M. ovipneumoniae* cells for 48 h and cell lysates were then harvested for Western blot analysis. Compared to the control, the phosphorylation of ERK1/2, JNK/c-Jun, and P38 was remarkably upregulated after treatments of CPS and bacterial cells (Figure 7), indicating an activation of MAPK pathway. Intriguingly, all three main pathways involved in the MAPK signaling, including the JNK, ERK1/2, and P38 were elevated in these epithelial cells upon CPS stimulation or* M. ovipneumoniae* infection. A pretreatment of NAC could reverse the effects. These results suggest a link and importance between MAPK signaling and CPS or* M. ovipneumoniae*-induced cell apoptosis in sheep bronchial epithelial cells.

### 3.8. CPS-Induced Cell Apoptosis Is Mainly due to an Activation of JNK and P38 MAPK Pathways in Sheep Bronchial Epithelial Cells

**Figure 8:**
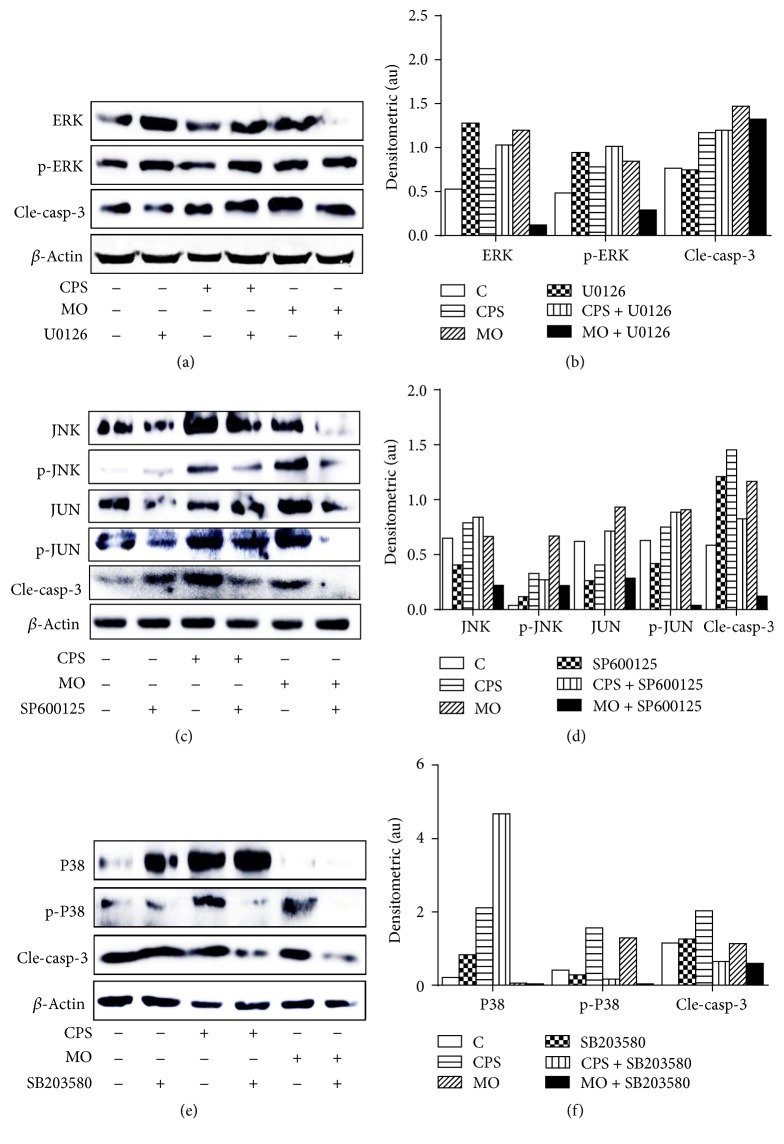
Involvements of MAPK signaling pathway in CPS-induced apoptosis in ALI cultures of sheep bronchial epithelial cells. Cells were pretreated with (a) U0126 (an ERK1/2 inhibitor, 10 *μ*M), (c) SP600125 (a JNK inhibitor, 20 *μ*M), or (e) SB203580 (a P38 inhibitor, 20 *μ*M) for 1 h, followed by exposure to CPS (100 ng/mL) or MO (MOI = 30) for 48 h. (a, c, e) Cell lysates were subjected to Western blotting analysis using indicated antibodies, showing that inhibitors of JNK (SP600125) and p38 (SB203580) but not ERK1/2 (U0126) significantly blocked the expression of CPS-induced apoptotic proteins. The immunoblots shown here were representative of three independent experiments with similar results. (b, d, f) Densitometric values for representative blots indicated proteins shown in (a), (c), and (e), respectively. *β*-actin was used as an internal control, and proteins were semiquantified by a densitometric analysis by calculating the fold of change of a protein of interest over respective *β*-actin.

To pinpoint how ERK1/2, JNK, and P38 are involved in CPS- or* M. ovipneumoniae-*induced apoptosis in ALI cultures of sheep bronchial epithelial cells, we further investigated impacts of U0126 (an ERK1/2 inhibitor), SP600125 (a JNK inhibitor), and SB203580 (a P38 inhibitor) on MAPK signaling in sheep bronchial epithelial cells in response to CPS stimulation or bacterial infection. To this end, cells were cultured in the presence or absence of U0126 (10 *μ*M), SP600125 (20 *μ*M), or SB203580 (20 *μ*M) for 1 h and then exposed to CPS/*M. ovipneumoniae* for an additional 48 h. Unexpectedly, a pretreatment of ERK inhibitor U0126 failed to inhibit the CPS*-*induced activation of ERK1/2 and caspase-3 (Figures 8(a) and 8(b)). In contrast, a pretreatment of either JNK signaling inhibitor SP600125 (Figures 8(c) and 8(d)) or p38 signaling inhibitor SB203580 (Figures 8(e) and 8(f)) showed a significantly downregulated CPS- and* M. ovipneumoniae-*induced phosphorylation of JNK/c-Jun or p38, with a remarkable decrease of the activity of caspase-3. Taken together, these results imply that CPS*-*induced apoptosis may be mainly attributed to an activation of JNK and p38, but not ERK1/2 of the MAPK pathways, although all three MAPK members including ERK1/2, JNK, and P38 can be activated by stimuli of CPS and* M. ovipneumoniae* in sheep bronchial epithelial cells.

## 4. Discussion

In the present study, we describe the isolation and purification of the capsular polysaccharide (CPS) of* M. ovipneumoniae* for the first time and substantially evaluate the effects and possible molecular mechanism in cell apoptosis of sheep bronchial epithelial cells. Our results demonstrate that the CPS had a cytotoxic effect of decreasing cell viability with an ability to induce ROS production, which in turn disrupted the integrity of mitochondrial membrane, along with a reduction of ΔΨ*m* and an increased expression of proapoptotic proteins. Mechanistically, the CPS-induced cell apoptosis was induced by mechanisms involved in caspase-dependent intrinsic and extrinsic apoptotic pathways. Moreover, the CPS-induced intrinsic cell apoptosis was ROS-dependent and mediated by MAPK signaling pathways. In this regard, the JNK and p38 but not the ERK signaling of the MAPK pathway might be the main intrinsic apoptotic pathways that contributed to the CPS-induced apoptosis in these cells.

Apoptosis is an active form of programmed cell death that plays a vital role in the maintenance and development of organism homeostasis [8, 9, 26]. It allows cells to undergo a highly regulated form of death in response to various proapoptotic stimuli. Evidence has demonstrated that ROS are generated from both mitochondria and NADPH oxidases [7, 27], which has been well defined to regulate various signal transduction pathways including inflammation and apoptosis. Thus, in this study we tentatively explored the potential mechanism that CPS induces apoptotic process* via* the generation of ROS in ALI cultures of sheep bronchial epithelial cells, and an elevated production of ROS was found in cells exposed to the CPS and* M. ovipneumoniae* bacteria cells.

It is well known that an activation of caspase represents a crucial step in the induction of apoptosis, and the cleavage of poly ADP-ribose polymerase (PARP) by caspase-3 is also considered to be one of the hallmarks of apoptosis [18]. FACS analysis showed an increased proportion of apoptotic cells in CPS- and* M. ovipneumoniae*-treated cells compared to untreated cells, which was consistent with the results from Western blotting, from which the cleaved-caspase-3 and cleaved-PARP1 were increased in both CPS- and* M. ovipneumoniae-*treated cells. Of particular interest, the caspase-3, one of the most widely studied and the most key apoptosis effectors [9], was strikingly activated following a stimulation of CPS and* M. ovipneumoniae* cells, thereby suggesting that CPS- and* M. ovipneumoniae-*induced cell toxicity were at least in part related to an apoptotic progression. In order to determine the effects of ROS in regulating apoptosis, a ROS scavenger, NAC (N-Acetyl-L-cysteine), was used to further clarify the underlying mechanism. Results showed that a pretreatment of NAC could dramatically downregulate the generation of ROS induced by CPS and* M. ovipneumoniae*, which resulted in an increase in CAT, SOD_2_, and GSS levels. Interestingly, cytotoxicity of CPS was still observed in treated cells even if NAC pretreatment could scavenge most of ROS production, suggesting that there may exist other proapoptotic mechanisms apart from the ROS-induced oxidative damage of epithelial cells. Collectively these data suggest that ROS might be an essential player in apoptosis of cells in response to* M. ovipneumoniae* infection; however, its precise role in CPS-induced apoptosis needs further investigation.

Mitochondria are a main generator of ROS, which play an essential role in the oxidative stress-triggered signaling pathway during the process of apoptosis [28–30]. The integrity of mitochondrial membrane serves as the basis for mitochondria function, which can be mainly regulated by different Bcl-2 family members including proapoptotic proteins, such as Bax, and antiapoptotic proteins, such as Bcl-2 and Bcl-xl [31, 32]. Previous studies have documented that Bax, the most important proapoptotic protein, can induce the translocation of cytochrome C from mitochondria to cytoplasm. The released cytochrome C subsequently leads to the release of apoptosis inducing factor (AIF), which in turn activates caspase cascades, finally resulting in cell apoptosis [33, 34]. In this signal cascade, caspases are key regulators in the apoptotic pathway, which generally consist of the upstream initiators, such as caspase-8, -9, and -10 and the downstream effectors such as caspase-3, -6, and -7 [9]. In line with this notion, we firstly confirmed that mitochondria were involved in CPS- and* M. ovipneumoniae*-induced cell apoptosis. The results showed that an exposure of CPS or an infection of* M. ovipneumoniae* could markedly upregulate the percentage of apoptotic cells compared to the control. In contrast, an elimination of ROS with NAC led a partial suppression of apoptotic effects induced by CPS and* M. ovipneumoniae*. Immunoblotting analysis further revealed that the occurrences of apoptosis induced by both CPS and* M. ovipneumoniae* were linked to an upregulation of proapoptotic protein Bax, cytochrome C, and AIF in ALI cells. Furthermore, a scavenging ROS by NAC could alleviate the CPS- and* M. ovipneumoniae*-induced mitochondrial damage, indicating that the intrinsic apoptosis pathway might be at least in part involved in the finally apoptotic death of ALI cells. Notably, inconsistent with results from other studies, the expression of Bcl-2 in the present study was evidently upregulated in response to CPS- or* M. ovipneumoniae* stimulation, suggesting that Bcl-2 might play a protective role in these cells in response to excessive oxidative stress, therefore rescuing cells from cell death [31]. Another possible reason might be that the Bax could antagonize the antiapoptotic effects of Bcl-2 and Bcl-xl* via* a binding to one another depending on a BH_3_ domain, thereby promoting apoptosis [35].

To further elucidate the role of CPS in* M. ovipneumoniae-*induced apoptosis, we examined the effects of CPS in FAS receptor-mediated extrinsic apoptotic pathway. Results showed that the expressions of FAS-associated proteins including FADD, FAS, FASL, caspase-8, and cleaved-caspase-8 were extremely upregulated in response to CPS and* M. ovipneumoniae* treatments. This effect could be completely inhibited by ROS scavenger NAC, indicating that an increased transcriptional activity of FAS-associated proteins was downstream of ROS signaling. The result was further confirmed by detecting the activation of caspase-8, an upstream initiator of caspase-3 involved in extrinsic apoptotic pathway [36, 37]. Interestingly, our results also showed that the CPS and* M. ovipneumoniae*-mediated extrinsic apoptotic signaling were much more activated relative to intrinsic apoptotic signaling suggesting that an extrinsic apoptotic signaling might contribute significantly to CPS-induced epithelial cell apoptosis in this study.

Given the fact that both intrinsic and extrinsic apoptotic pathways are involved in the CPS of* M. ovipneumoniae*-induced apoptosis in ALI cultures of sheep bronchial epithelial cells, the underlying mechanisms by which CPS inhibited ALI cells were thus further explored. The MAPKs signaling is believed to be involved in the regulation of several signal transduction pathways including cell proliferation, migration, differentiation, autophagy, and apoptosis [8, 38]. MAPKs are evolutionarily conserved Ser/Thr protein kinases that are divided into at least three distinct groups in mammals, including the extracellular signal regulated kinase (ERK), the Jun N-terminal kinases (JNK), and the P38 MAPKs [39]. Mechanistically, upon an activation of this signaling cascade, ERK usually plays an antiapoptotic role, while JNK and P38 exert proapoptotic effects during an apoptotic process [40, 41]. In this context, the dynamic equilibrium between the opposing effects of ERK and JNK/P38 MAPK may be critical in determining whether cells survive or undergo apoptosis. Mounting evidences have demonstrated that ROS-induced apoptotic cell death is mainly mediated by JNK and P38 MAPK activation [42], both of which initiate mitochondria-dependent apoptosis through a caspases signaling pathway, partially owing to an enhanced transcription of proapoptotic genes, such as Bax [43, 44]. In agreement with these notions, we showed that both CPS and* M. ovipneumoniae* stimuli resulted in an activation of all the three MAPK family members, but only the JNK and p38 pathways but not the ERK1/2 signaling participated in CPS-induced apoptosis. This observation is of particular mentioning to importance due to the fact that an inhibitor of ERK1/2 signaling is incapable of reducing the activation of caspase-3, indicating that CPS-induced apoptosis can be independent of ERK1/2 signaling and can progress alternatively through the JNK and p38 pathways. Of note, this observation was different from the* M. ovipneumoniae* bacteria-induced cell apoptosis determined in our recent study, in which* M. ovipneumoniae* was demonstrated to induce the cell apoptosis through an ERK-mediated MAPK pathway [15]. The discrepancy suggested distinct pathogenic roles of CPS of* M. ovipneumoniae*, as compared with its parent pathogen bacteria cells.

**Figure 9:**
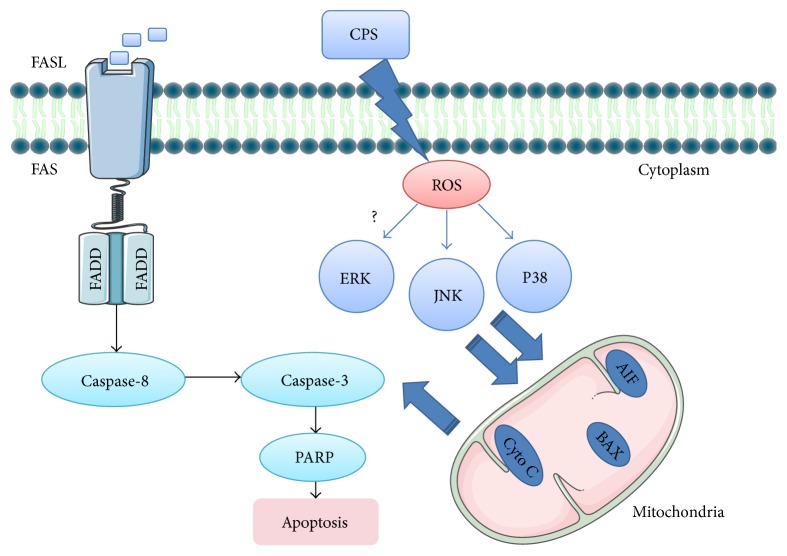
Scheme showed a possible mechanism of CPS-induced caspase-dependent apoptosis in ALI cultures of sheep bronchial epithelial cells. ROS-mediated JNK/P38 MAPK and FAS/FASL signaling pathways may be the main routes contributing to CPS-induced apoptosis.

The JNK is another key regulator in oxidative stress-induced apoptosis, as well as in the autophagy induction [45–48], and ROS have been reported to be a strong activator of JNK signaling and play a crucial role in JNK-dependent apoptosis and autophagy regulation [49]. Although our results showed that CPS- and* M. ovipneumoniae-*induced activation of JNK were accompanied by ROS production, it is noteworthy that NAC failed to alleviate the* M. ovipneumoniae*-induced phosphorylation of JNK, which contradicted the result of CPS, suggesting that ROS may be not the sole upstream activator of JNK signaling pathway in response to a* M. ovipneumoniae* infection. One possible explanation is that the activation of JNK participated in a crosstalk between apoptosis and autophagy triggered by* M. ovipneumoniae via* promoting autophagy and suppressing apoptosis in the presence of NAC. Consistently, both CPS and* M. ovipneumoniae* could induce an evident PARP-1 activation that was suggested to cause a prosurvival autophagy* via* the AMPK-mTOR signaling pathway [50]. This result therefore indicates that CPS- and* M. ovipneumoniae*-induced oxidative stress may be involved in the autophagy induction. As a consequence, it is reasonable to hypothesize that CPS- and* M. ovipneumoniae*-treatment simultaneously induce the progress of apoptosis and autophagy in ALI cultures of sheep bronchial epithelial cells, which requires further investigations. Taken together, we hypothesize that the ROS-mediated JNK/P38 MAPK signaling pathways may be one of the main routes contributing to CPS-induced apoptosis. These findings thus provide insight into the molecular pathways that lead to apoptosis of* M. ovipneumoniae-*infected respiratory tract epithelia (Figure 9).

## 5. Conclusions

Collectively, this study reveals that ROS-regulated activation of JNK and p38 signaling may play key roles in sheep airway epithelial cells upon* M. ovipneumoniae* infection. Moreover, the capsular polysaccharide (CPS) of* M. ovipneumoniae* serves as an important virulence factor, contributing to* M. ovipneumoniae-*induced apoptosis through both ROS-dependent-intrinsic and extrinsic apoptotic pathways. Our findings underline that CPS of* M. ovipneumoniae* may be exploited for acceleration of* M. ovipneumoniae-*induced oxidative stress and consequent disruption of homeostasis. Besides, considering that CPS may participate in autophagy and necrosis, whose activation in a ROS/JNK-dependent manner may serve as other critical mechanisms for cell death, whether CPS/*M. ovipneumoniae* is involved in above two processes remains unknown. Thus, further study on the regulation of autophagy and necrosis will help us establish a clearer understanding of the mechanism of CPS- and* M. ovipneumoniae-*induced cell death in sheep respiratory epithelial cells.
